# Detection of* Folliculin* Gene Mutations in Two Chinese Families with Birt-Hogg-Dube Syndrome

**DOI:** 10.1155/2017/8751384

**Published:** 2017-07-12

**Authors:** Lv Liu, Kai Yang, Xiang Wang, Zhihui Shi, Yifeng Yang, Yu Yuan, Ting Guo, Xiaocui Xiao, Hong Luo

**Affiliations:** ^1^Department of Respiratory Medicine, Diagnosis and Treatment Center of Respiratory Disease, The Second Xiangya Hospital, Central South University, Changsha, Hunan 410011, China; ^2^Department of Cardiothoracic Surgery, The Second Xiangya Hospital, Central South University, Changsha 410011, China; ^3^Department of Thoracic Surgery, The Second Xiangya Hospital, Central South University, Changsha 410011, China

## Abstract

Birt-Hogg-Dube syndrome (BHD, OMIM#135150) is a rare disease in clinic; it is characterized by skin fibrofolliculomas, pulmonary cysts with an increased risk of recurrent pneumothorax, renal cysts, and renal neoplasms. Previous studies have demonstrated that variants in* folliculin* (*FLCN*, NM_144997) are mainly responsible for this disease. In this research, we enrolled two BHD families and applied direct sequencing of* FLCN* to explore the genetic lesions in them. Two* FLCN* mutations were identified: one is a novel deletion variant (c.668delA/p.N223TfsX19), while the other is a previously reported insertion mutation (c.1579_1580insA/p.R527QfsX75). And the pathogenicity of both variants was confirmed by cosegregation assay. Bioinformatics analysis showed that c.668delA may lead to functional haploinsufficiency of* FLCN* because mRNA carrying this mutation exhibits a faster degradation rate comparing to the wild type. Real-time qPCR also confirmed that the mRNA level of* FLCN* expression in the proband was decreased significantly compared with the controls, which may disrupt the mTOR pathway and lead to BHD. The insertion mutation (c.1579_1580insA) was predicted to cause a prolonged amino acid sequence of FLCN. The present identification of two mutations not only further supports the important role of tumor suppressor FLCN in BHD and primary spontaneous pneumothorax, but also expands the spectrum of* FLCN* mutations and will provide insight into genetic diagnosis and counseling of families with BHD.

## 1. Introduction

Birt-Hogg-Dube syndrome (BHD, OMIM#135150) is a rare autosomal dominant disorder mainly featured by skin fibrofolliculomas, pulmonary cysts with an increased risk of recurrent pneumothorax, renal cysts, and renal neoplasms [1, 2]. The renal neoplasms in BHD patients are predominantly presented hybrid oncocytic/chromophobe and subtype followed by the clear cell renal carcinomas [3]. And more than 85% of patients show the benign dermatological papules phenotypes, which usually do not appear before the age of 20 [4, 5]. Currently, there is no international consensus for surveillance of BHD patients.

Previous studies have demonstrated that* folliculin* (*FLCN*, NM#144997), located at 17p11.2, was the main causative gene responsible for BHD [6, 7]. There is a rapid growing interest for clinical diagnosis of the* FLCN* mutations that underlie BHD since the discovery of the first variant in 2002 [8]. To date, approximately 169 variants of* FLCN* have been reported in BHD or isolated pneumothorax and renal carcinoma patients [2]. According to the data from LOVD database (https://grenada.lumc.nl/LOVD2/shared1/home.php?select_db = FLCN), frameshift mutation and missense nutation are the common variants in BHD patients.

In this study, we investigated two clinically characterized families with BHD and primary spontaneous pneumothorax (PSP). An autosomal dominant inheritance pattern has been observed in these two families. By applying direct sequencing of* FLCN*, we identified a novel deletion mutation (c.668delA/p.N223TfsX19) and a previously reported insertion mutation (c.1579_1580insA/p.R527QfsX75) [9–11] of* FLCN* that might underlie these two families.

## 2. Material and Methods

### 2.1. Subjects

The Review Board of the Second Xiangya Hospital of the Central South University has approved this research. All family members gave written informed consent. Blood was collected from the probands and their family members; subjects were examined by CT testing and B ultrasonic testing. Renal tissues were collected from surgery.

### 2.2. DNA Extraction

Genomic DNA was prepared from peripheral blood of the patients and all other participants using a DNeasy Blood & Tissue Kit (Qiagen, Valencia, CA) as we have described [12].

### 2.3. Mutation Sequencing

The entire coding regions, including the flanking intronic sequences of* FLCN* (NM_144997), were amplified with polymerase chain reaction (PCR; primer sequences will be provided upon requests). Sequences of the PCR products were determined by the ABI 3100 Genetic Analyzer (ABI).

### 2.4. Sequence Alignments and Bioinformatic Prediction

The multiple* FLCN* protein sequences across mammals were aligned using the program MUSCLE (version 3.6). The PolyPhen-2 (polymorphism phenotyping), SIFT (Sorting Intolerant From Tolerant), and MutationTaster programs were used to predict the effects of mutations on the function of the proteins [13].

### 2.5. RNA Extraction and Real-Time qPCR

Total RNA was extracted by the PureLink® RNA Mini (Thermo Fisher Scientific, #12183025) from the renal tissues in surgery (in proband 2 a surgical excision of a tumor from the kidney was performed; the control was from other patient tumor borderline tissues without* FLCN* mutation). The cDNA was synthesized from a total of 1 *μ*g of RNA using the RevertAid First Strand cDNA Synthesis Kit (Thermo Fisher Scientific, #K1621) with oligo (dT) primers. Real-time qPCR reactions were carried out in Fast 7500 Real-Time PCR Systems (Applied Biosystems) using Maxima SYBR Green/ROX qPCR Master Mix (2x) (Thermo Fisher Scientific, #K0221). And 2^(−ΔΔCt)^ was used to analyze the comparative* FLCN* mRNA expression levels between mutation group and healthy group. Each assay was performed in five independent tests. The data were analyzed by unpaired two-tailed *t*-tests using GraphPad Prism V.5 software (V.5.0). And the sequences of PCR primers will be provided upon request.

## 3. Results

### 3.1. Clinical Features

**Figure 1:**
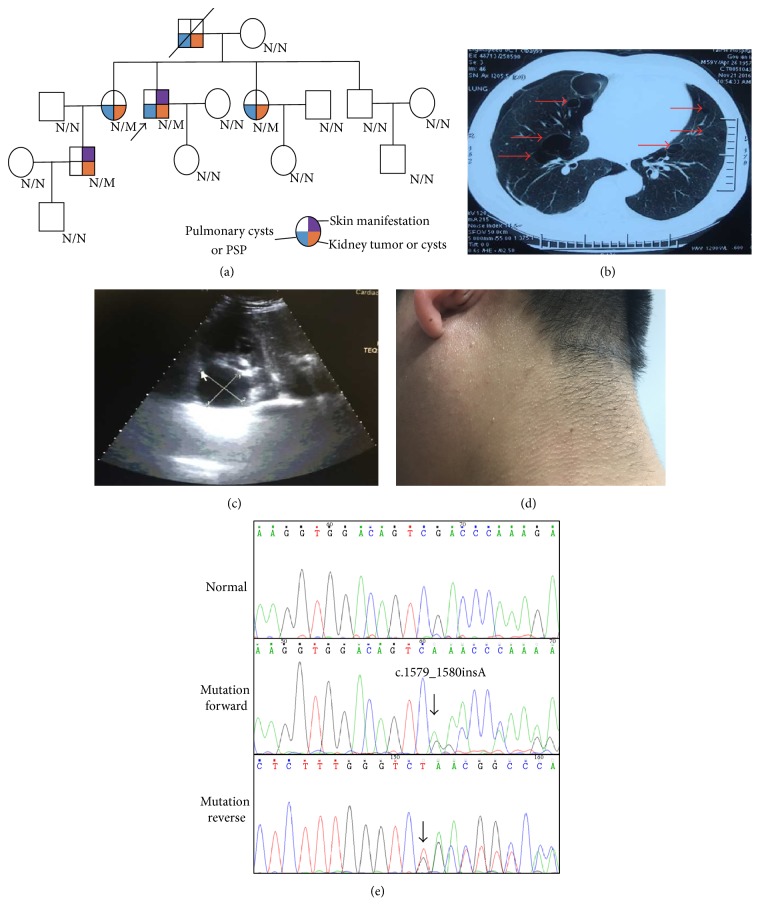
The clinic and genetic data of Family 1. (a) Pedigree of Family 1 affected with BHD. Squares indicate male family members; circles, female members; N/N normal type; N/M mutation type; arrow, proband. (b) Lung CT testing result of proband in Family 1. Red arrows indicate multiple pulmonary cysts. (c) Kidney B ultrasonic testing results of proband in Family 1. The low echoic area indicates multiple renal cysts. (d) Skin eruptions in the posterior neck of proband in Family 1. (e) Sequencing results of the* FLCN* mutation in Family 1. Sequence chromatogram indicates an A insertion in nucleotide 1579.

**Figure 2:**
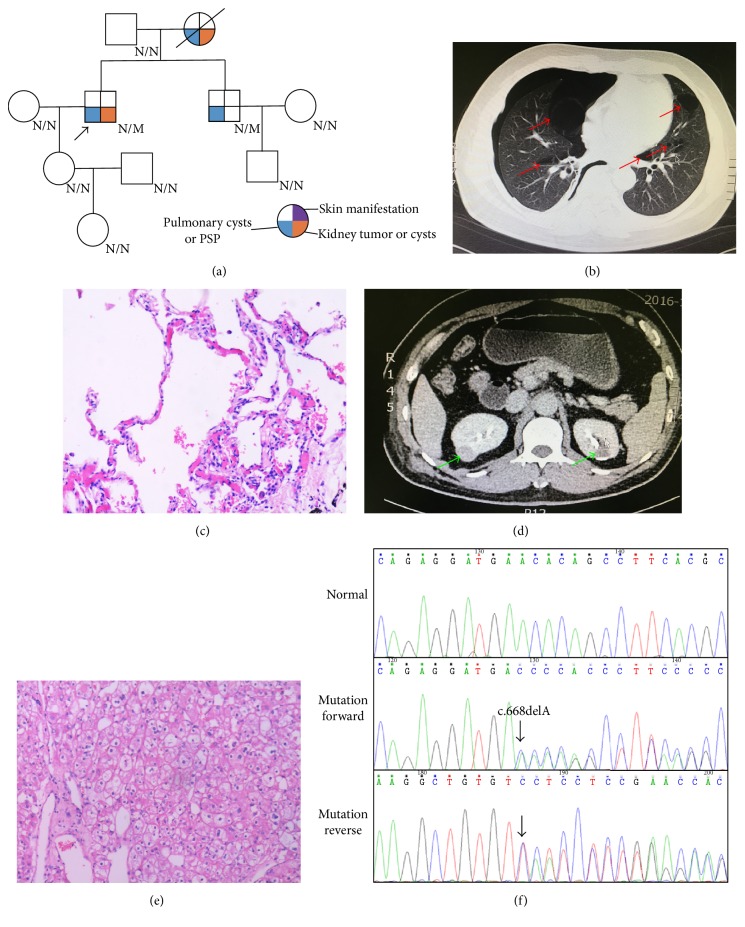
The clinic and genetic data of Family 2. (a) Pedigree of Family 2 affected with BHD. Squares indicate male family members; circles, female members; N/N normal type; N/M mutation type; arrow, proband. Pathological image of the renal mass. (b) Lung CT testing result of proband in Family 2. Red arrows indicate multiple pulmonary cysts. (c) Pathological image of the lung tissue. (d) Kidney contrast-enhanced CT testing result of proband in Family 2. Green arrows indicate renal tumor (right) or renal cyst (left). (e) Pathological image of the renal tumor tissue. (f) Sequencing results of the* FLCN* mutation in Family 2. Sequence chromatogram indicates an A deletion in nucleotide 668.

Two families from Central South China (Hunan province) were presented in this study. In Family 1, proband 1, a 59-year-old male suffered from a repeated bilateral pneumothorax sequentially. CT scan reveals an expression of pulmonary cysts (Figures 1(a) and 1(b)), while B-ultrasound ensures multiple renal cysts with liver cysts (Figure 1(c)). In addition, many skin eruptions can also be seen in his posterior neck (Figure 1(d)). And he was diagnosed as suspected BHD. Family history investigation showed another 4 members who also shared similar symptoms (Figure 1(a)). In Family 2, proband 2, there was a 46-year-old male. He was admitted in the hospital due to primary spontaneous pneumothorax (PSP) and right renal neoplasms (Figures 2(a), 2(b), and 2(c)). Contrast-enhanced CT scan reveals an expression of left kidney cysts and liver cysts (Figure 2(d)). The pathological section of his lung pathology presents pulmonary cyst (Figure 2(b)), while his renal tissue accords with renal cell carcinoma (Figure 2(e)). His mother also suffered from PSP and renal neoplasms while his brother only presents PSP. All members in F2 exhibited absence of skin fibrofolliculomas and were also suspected as a BHD pedigree (Figure 2(a)).

### 3.2. Genetic Analysis Identified a Novel Segregating Mutation in* FLCN*

We investigated these two families by Sanger sequencing. In Family 1, a reported insertion mutation (c.1579_1580insA/p.R527QfsX75) in exon 14 of* FLCN* was identified in proband 1 (Figure 1(e)). Cosegregation analysis found that all the patients in Family 1 harbor this mutation (Table 1). In Family 2, a novel deletion variant in* FLCN*, c.668delA located in exon 7, was detected and cosegregated with the affected family members (Table 1, Figure 2(f)). This novel frameshift mutation (c.668delA/p.N223TfsX19), resulting in a premature stop codon at position 242 of the* FLCN *gene, was not found in our 200 control cohorts. In addition, this newly identified mutation was also not presented in the dbSNP and Exome Variant Server database (http://evs.gs.washington.edu/EVS/).

### 3.3. The Novel Segregating Mutation Decreases the mRNA Expression Level of* FLCN*

**Figure 3:**
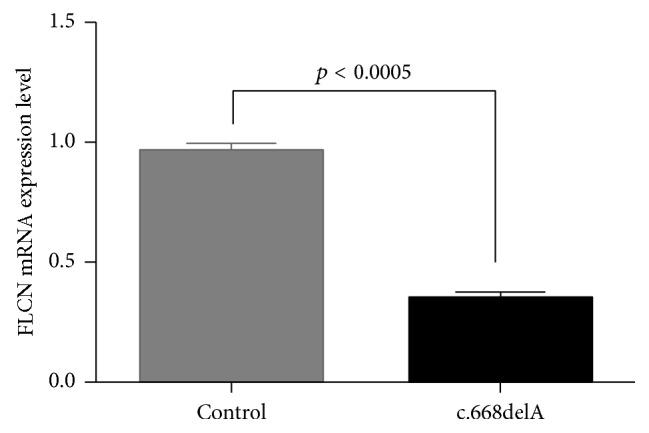
RNA expression of* FLCN* in affected individual and controls. Mean expression (±SEM) of* FLCN* in affected individual and control measured by real-time qPCR.

The bioinformatics program MutationTaster predicated that both mutations were disease causing. The novel frameshift mutation (c.668delA/p.N223TfsX19) of* FLCN* may lead to a premature stop codon in exon 7. According to nonsense-mediated mRNA decay theory [14], the levels of* FLCN* mRNA expression in affected patients may decrease. We then isolated the mRNA from the renal tissues in surgery. Real-time qPCR regarded the control levels of mRNA in* FLCN* as “1.” The results revealed that the level of* FLCN* mRNA expression in proband 2 was decreased significantly compared with the controls (*p* < 0.0005) (Figure 3).

## 4. Discussion

In this study, we employed Sanger sequencing to explore the genetic factors of two BHD families. A reported variant (c.1579_1580insA/p.R527QfsX75) of* FLCN* was identified in Family 1, while in Family 2, we detected a novel* FLCN* frameshift mutation (c.668delA/p.N223TfsX19) (Table 1). The reported mutation (c.1579_1580insA) was located in the C-terminus of FLCN and may affect the structure and function of this protein. The novel mutation (c.668delA) may lead to functional haploinsufficiency of* FLCN*, which was confirmed by real-time qPCR.

*FLCN* encodes protein FLCN, a tumor suppressor protein which can regulate cellular activities by interacting with FNIP1 and AMP-activated protein kinase (AMPK), a key molecule for energy sensing that negatively regulates mTOR activity. Previous studies have revealed that C-terminus of FLCN and amino acids 300 to 1166 of FNIP1 were needed for optimal FLCN-FNIP1 binding, which helped the localization to the cytoplasm [15, 16]. In our study, the novel frameshift mutation (c.668delA/p.N223TfsX19) may lead to functional haploinsufficiency of FLCN, which may fail to regulate mTOR activity and lead to BHD. In addition, as a tumor suppressor protein, the novel mutation may decrease the levels of FLCN protein and induce the renal tumor [2, 8].

The mutation c.1579_1580insA was first reported in East Asia population [9]; then it was also detected in Taiwanese and Indian [10, 11]. This mutation was predicted to cause a prolonged amino acid sequence which changed the C-terminal of the FLCN protein since the 527 site. This mutation is predicted to disturb FLCN interacting with FNIP and AMPK, resulting in mTOR pathway disorder [8]. In our study, both proband 1 and proband 2 were affected by pneumothorax, but the phenotypes of kidney disease were different. Proband 1 showed renal cysts while proband 2 present an obvious kidney neoplasm. These differences may be due to the types of different mutations and genetic heterogeneity [17].

Mutations in* FLCN* are highly capable of leading to BHD. In this family, several lines of evidences support the notion that the novel mutation (c.668delA/p.N223TfsX19) in* FLCN* is related causally to BHD: (1) variation in this site was not found in 200 unrelated subjects, dbSNP and Exome Variant Server database, suggesting that it is not a common polymorphism; (2) no other meaningful mutations were found in the rest of the* FLCN* and bioinformatics programs predicted this mutation is deleterious; (3) this base change was not found in other siblings who did not present BHD or isolated pneumothorax and renal carcinoma; (4) the mutation may lead to functional haploinsufficiency of FLCN which was confirmed by real-time qPCR; (5) in addition, there are several mutations (c.671_672delCA, c.769_771delTCC, and c.715C>T) located in the similar domain of the FLCN which have been identified to be related to the BHD or isolated pneumothorax and renal carcinoma [18–20].

## 5. Conclusion

In conclusion, we identified a known insertion mutation (c.1579_1580insA/p.R527QfsX75) and a novel deletion variation (c.668delA/p.N223TfsX19) of* FLCN* in two BHD families, respectively. The insertion mutation may cause a prolonged amino acid sequence of FLCN. The novel deletion variation may lead to functional haploinsufficiency of* FLCN*, which was confirmed by real-time qPCR. The present identification of these two mutations not only further supports the important role of tumor suppressor FLCN in BHD and PSP, but also expands the spectrum of* FLCN* mutations and will provide insight into genetic diagnosis and counseling of families with BHD.

## Figures and Tables

**Table 1:** The clinic and genetic summary of these two families.

Family ID	Phenotype in family members				Mutation analysis				
	Clinically affected	Skin eruptions	Renal tumors/cysts	Lung cysts/PSP	Gene	Exon	Nucleotide	Protein	Mutation type
1	Proband	Yes	Yes	Yes	*FLCN*	14	c.1579_1580insA	p.R527QfsX75	Insertion
1	Father	No	Yes	Yes					
1	Elder sister	No	Yes	Yes					
1	Sister	No	Yes	Yes					
1	Nephew	Yes	Yes	No					

2	Proband	No	Yes	Yes	*FLCN*	7	c.668delA	p.N223Tfs*∗*19	Deletion
2	Mother	No	Yes	Yes					
2	Brother	No	No	Yes					
